# Learning from irregularly sampled data for endomicroscopy super-resolution: a comparative study of sparse and dense approaches

**DOI:** 10.1007/s11548-020-02170-7

**Published:** 2020-05-15

**Authors:** Agnieszka Barbara Szczotka, Dzhoshkun Ismail Shakir, Daniele Ravì, Matthew J. Clarkson, Stephen P. Pereira, Tom Vercauteren

**Affiliations:** 1 Wellcome/EPSRC Centre for Interventional and Surgical Sciences, University College London, London, UK; 2 Department of Surgical & Interventional Engineering, King’s College London, London, UK; 3 Centre for Medical Image Computing (CMIC), Department of Computer Science, University College London, London, UK; 4 UCL Institute for Liver and Digestive Health, University College London, London, UK

**Keywords:** Nadaraya–Watson kernel regression, pCLE reconstruction, Super-resolution, CNN

## Abstract

**Purpose:**

Probe-based confocal laser endomicroscopy (pCLE) enables performing an optical biopsy via a probe. pCLE probes consist of multiple optical fibres arranged in a bundle, which taken together generate signals in an irregularly sampled pattern. Current pCLE reconstruction is based on interpolating irregular signals onto an over-sampled Cartesian grid, using a naive linear interpolation. It was shown that convolutional neural networks (CNNs) could improve pCLE image quality. Yet classical CNNs may be suboptimal in regard to irregular data.

**Methods:**

We compare pCLE reconstruction and super-resolution (SR) methods taking irregularly sampled or reconstructed pCLE images as input. We also propose to embed a Nadaraya–Watson (NW) kernel regression into the CNN framework as a novel trainable CNN layer. We design deep learning architectures allowing for reconstructing high-quality pCLE images directly from the irregularly sampled input data. We created synthetic sparse pCLE images to evaluate our methodology.

**Results:**

The results were validated through an image quality assessment based on a combination of the following metrics: peak signal-to-noise ratio and the structural similarity index. Our analysis indicates that both dense and sparse CNNs outperform the reconstruction method currently used in the clinic.

**Conclusion:**

The main contributions of our study are a comparison of sparse and dense approach in pCLE image reconstruction. We also implement trainable generalised NW kernel regression as a novel sparse approach. We also generated synthetic data for training pCLE SR.

**Electronic supplementary material:**

The online version of this article (10.1007/s11548-020-02170-7) contains supplementary material, which is available to authorized users.

## Introduction

Probe-based confocal laser endomicroscopy (pCLE) is a recent optical fibre bundle-based medical imaging modality with utility in a range of clinical indications and organ systems, including gastrointestinal, urological and respiratory tracts [5].

The pCLE probe relies on a coherent fibre bundle comprising many (>10k) cores that are irregularly distributed across the field of view (FoV). The nature of image acquisition through coherent fibre bundles constitutes a source of inherent limitations in pCLE, having a direct, negative impact on the image quality. The raw data that the pCLE devices produce therefore remain challenging to use for both clinicians and computerised decision support systems.

Raw pCLE are distorted by a few artefacts such as a honeycomb pattern and so need to be corrected before reconstruction. During calibration and restoration, the raw image is transformed into a vector of corrected fibre signals and their locations in the space of the fibre FoV [12]. The irregular sampling domain of the signals can be accurately discretised as a set of locations in an over-sampled regular grid and then interpolated.

Existing pCLE image reconstruction approaches typically use Delaunay triangulation to linearly interpolate irregularly sampled signals onto a Cartesian grid [22]. These interpolation methods allow to reconstruct the Cartesian image, yet do not enhance image quality nor take into account any prior knowledge of the image space except for regularisation-related properties. Moreover, they are themselves prone to generating artefacts, such as triangle edge highlights or additional blur [22].

Deep learning (DL) has been widely used particularly in medical image analysis [4] due to efficient formulations behind it [15], although there are still some challenging issues such as over-fitting [2] and the unnatural fit for sparse inputs. It was shown that state-of-the-art CNN-based single-image super-resolution (SISR) techniques can improve the quality of pCLE images [18]. A potential limitation in the current CNN approaches is that the analysis starts from already reconstructed pCLE images, including reconstruction artefacts.

There are a few research works focusing on allowing sparse data as CNN input [3, 6, 20]. Generalising the conclusion from these studies to pCLE hints to the intuition that applying CNNs directly to irregularly sampled pCLE data may not be trivial. We study a suitability of three DL approaches applied either directly to the irregularly sampled or reconstructed Cartesian images, respectively. The main focus of this work is to compare baseline pCLE image reconstructions obtained from classical interpolation methods to these dedicated DL approaches.

For the study, we design a novel trainable convolutional layer called an NW layer, which integrates Nadaraya–Watson (NW) kernel regression [16] into the DL framework, allowing a principled handling of irregularly sampled data in the neural network. To the best of our knowledge, we are the first to propose using NW kernel regression embedded in a CNN framework. We make use of it to design a network for medical image super-resolution reconstruction from irregularly sampled pCLE signals. It is also the first work delivering a head-to-head comparison of sparse and Cartesian approaches for pCLE SISR.

## Related work

*pCLE image reconstruction* The pCLE image reconstruction algorithm in current clinical use is based on Delaunay triangulation. It yields sharp images, but contains triangulation artefacts [21]. Image reconstruction from sparse signals has been widely studied. Specifically, in the context of pCLE, Vercauteren et al. [22] implemented reconstruction from scattered pCLE data with NW kernel regression using handcrafted Gaussian weighting kernels. They demonstrated that the method efficiently reconstructs pCLE images and mosaics, at the price of some additional blur in comparison with Delaunay reconstruction.

*pCLE super-resolution* It has been shown that CNNs could effectively improve pCLE quality. In the study [18], researchers compared the performance of state-of-the-art SISR networks in reconstructing SR pCLE images. Due to the lack of ground truth high-resolution (HR) images, they used mosaicing to estimate synthetic HR images and simulated pCLE signal loss to create synthetic low-resolution (LR) images. They confirmed both quantitatively and qualitatively that CNNs trained on the synthetic data can improve the pCLE image quality.

Another work showing a proof of concept for SR reconstruction uses DenseNet trained on pairs of LR and HR patches [7]. The LR endomicroscopy was simulated by bi-cubic down-sampling of HR confocal laser endomicroscopy (CLE). These CLE images are generated by the Pentax EC-3870FK, and they are not affected by same distortions as pCLE is, because the Pentax device does not use a fibre bundle as an imaging guide. They prove that their DL solution outperforms classical interpolation by recovering HR details and reduces pixelation artefacts when used to super-resolve synthetic LR images.

In the absence of HR pCLE images, also unsupervised blind super-resolution has been proposed. The work contributes an adversarial network with cycle consistency for pCLE [17]. However, all these works use reconstructed Cartesian pCLE and CLE images as input to the networks.

*Sparse CNN inputs* While convolution layers are widely used, they have been identified as suboptimal for dealing with sparse data [20]. Much of the available literature on exploring sparsity in the context of CNN input deals with the irregular data in an intuitive but ad hoc way: non-informative pixels are assigned zero, creating an artificial Cartesian image. For example, Li et al. [13] used that technique and assigned the missing points zeros on a LR image. A similar workaround is to use an additional channel to encode the validity of each pixel like in Kohler et al. [11]; they passed a binary mask to the network. These solutions suffer from the redundancy in image representation due to spurious data being fed to the convolutional layers.

Uhrig et al. [20] proposed a convolutional layer which jointly processes sparse images and sparse masks to achieve sparsity invariant CNNs. Their sparse layer is designed to account for missing data during the convolution operation by modelling the location of data points with the use of a mask. This is achieved by convolving the mask with a constant kernel of ones while optimising the solution through convolving the sparse image with trainable kernels.

Following the success of sparse CNNs, Hua et al. [6] proposed to implement normalised convolution, as an extension of sparse convolution. They showed that using shared positive kernels for convolution with both an image and a mask is beneficial for upsampling depth maps. In both works, the information on sparsity is propagated to consecutive layers by the binary mask.

A demonstrated improvement in the proposed solutions is to use soft certainty maps, rather than propagating binary masks [3]. These maps are produced by updating the mask with the convolution. This method worked well in a guided depth upsampling task and uses both RGB data and LiDAR to reconstruct depth maps.

## Data generation methodology for the comparative study

Since common image quality assessment (IQA) relies on ground truth images used as a reference in metrics such as the peak signal-to-noise ratio (PSNR), the lack of ground truth high-resolution pCLE images makes it difficult to evaluate and compare the quality of SR reconstructions.

To address the lack of the HR pCLE, the authors in [18] proposed to use a first-generation SR method—an offline mosaicing—to simulate HR endomicroscopy. They used mosaics as a source of HR content; Unfortunately, these mosaics are not perfect enough estimate of HR images. The mosaicing resolves SR image from utilising overlap of video frames, and therefore, it suffers from miss-registration artefacts, and not a uniform overlay of the frames, which cause nonuniform contribution of SR resolving capabilities of the mosaicing on the entire surface of the SR image. The mosaicing is also time-consuming, making it not applicable to the real-time workflow of pCLE.

**Fig. 1:**
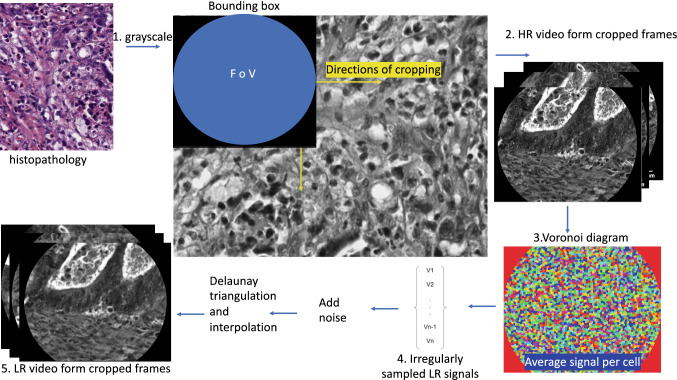
Illustration of the simulation for creation of synthetic data. Histological images are transformed to synthetic endomicroscopy

In this work, similar to [18], we used a triangulation-based reconstruction algorithm to simulate synthetic HR and LR endomicroscopy. However, in contrast to [18], we took advantage of the availability of histopathological images as a source of HR signals instead of using imperfect mosaics. During the diagnostic process, histopathological images play a similar role to pCLE. Since histopathological images are acquired with a digital camera, histology does not suffer from the problems created by irregularly distributed fibre signals. Thus, histopathological images meet the criteria of HR signal source and serve the role of synthetic ground truth in our synthetic data set. The simulation pipeline is illustrated in Fig. 1.

*Synthetic HR pCLE* In the first part of the simulation, we transformed RGB HR histological images into greyscale HR pCLE-like videos. The simulation starts with transforming RGB images into greyscale images. Next, we randomly selected original pCLE videos from the Smart Atlas [1], retrieved information on a bundle FoV and fibres locations for each video, and we matched randomly pCLE metadata with the histological images. To crop pCLE-like frames from the greyscale image, we were moving a bounding box of the fibre’s FoV from left to right, and from top to bottom in the image, with step size equal to half of the bounding box size. These pCLE-like frames were stacked to create the synthetic HR pCLE video sequence.

*Irregularly sampled synthetic LR pCLE* To simulate LR pCLE videos, we used the physically inspired pCLE-specific downsampling presented in [18]. In our case, the sources of irregular signals for physically-inspired pCLE-specific downsampling are HR synthetic pCLE videos. They are rich in high frequencies and pixel-level details.

For every synthetic HR pCLE, we used the given associated fibres location to build Voronoi diagrams with each fibre in the centre of the Voronoi cell. Every cell corresponds to the one fibre signal, yet cell space covers several pixels around the fibre on HR image. Thus, to simulate signal loss all HR pixels in that cell are averaged, and the average cell signal is used as a new LR fibre signal. The synthetic LR pCLE irregularly sampled images together with their associated given fibre position metadata may then be reconstructed as explained in “Irregularly sampled pCLE image reconstruction methods” section or used as input to a sparse super-resolution approach as detailed in “Learning-based super-resolution approaches” section.

## Irregularly sampled pCLE image reconstruction methods

**Fig. 2:**
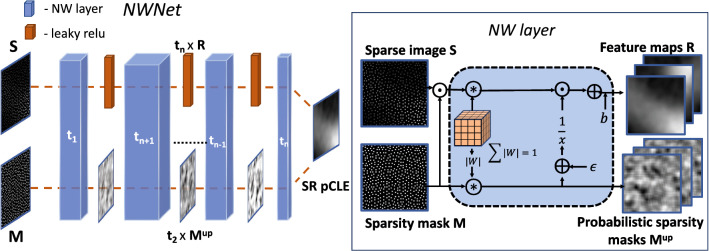
Graphical representation of NWNet framework (left) and NW layer (right)

*Irregularly sampled data as sparse image* Irregularly sampled data can be represented, with an arbitrary approximation quality, on a fine Cartesian grid as the sparse artificial Cartesian image *S*. Typically sparse image represents the sparse image space, where all non-informative pixels are set to 0. The pCLE sparse image is depicted in Fig. 2; we can see fibre signals (informative pixels), surrounded by non-informative space of zeros.

*Input masks* The information about the sampling grid is known from the nature of the acquisition. The position of the fibre signal in the Cartesian image corresponds to the position of the fibre within the bundle, which is given explicitly by a design of the fibre bundle and is provided with high accuracy by the manufacturer. We define pCLE mask *M* to be a representation of the position of the fibres in the Cartesian image space and represent it as a binary map. Intuitively, the image sparsity is encoded by ones and zeros for the informative and non-informative pixels, respectively, as a binary mask *M* of shape *S*. A sample *M* and its corresponding *S* are depicted in Fig. 2.

*Cartesian image reconstruction* To reconstruct sparse images to Cartesian images, the missing information is typically interpolated. This also means that the reconstructed images are over-sampled, and only a subset of the pixels carry information [18].

Typically, pCLE noise is interpolated onto the reconstructed image from noisy signals. To achieve that, we simulate pCLE noise, by adding it to the new fibre signal, before the interpolation step. We add multiplicative and additive Gaussian noise to mimic a calibration imperfection and an acquisition noise, respectively.

Synthetic LR pixels with added noise, obtained in the last step of data generation pipeline, are used as the irregularly sampled signals and reconstructed to the synthetic noisy Cartesian LR image using one of the baseline reconstruction approaches discussed hereafter. The synthetic LR pCLE has the size of the HR image, yet it is characterised by the lower image quality, noise, and reduced content of information due to simulated signal loss. Thanks to simulating signal distribution through the geometrical position of the fibres in the bundle, we simulate synthetic endomicroscopy as similar to real pCLE characterised by typical triangulation artefact and noise patterns.

The Cartesian images are reconstructed with our first baseline method, which is currently used in clinical practice. This reference reconstruction method is based on linear interpolation and Delaunay triangulation [22]. We also provide comparison to reconstructions obtained using NW kernel regression with a crafted single Gaussian kernel [21].

## Learning-based super-resolution approaches

We exploit the irregularly sampled pCLE data for image reconstruction tasks with CNNs. We compare state-of-the-art dense and sparse approaches for handling image sparsity by CNNs in application to pCLE image reconstruction with classical pCLE reconstruction methods.

*Dense approach* Reconstructed by Delaunay-based interpolation Cartesian image as input to CNN, the reconstructed pCLE images can be treated as any natural image and input to CNN directly. There is yet redundancy in the information at this image and strong prior for the network. Additionally, a convolutional layer is defined on the Cartesian grid and considers all image pixels as equally important regardless of their position.

*Sparse approach* A sparse image *S* is processed with a standard Cartesian CNN. *S* encapsulates both informative and non-informative pixels, and there is no explicit knowledge about the sampling pattern being fed to CNN. In that case, the network has to learn not only the mutual relations of informative pixels for the super-resolution task, but also handle the pixel sparsity. This dual role of the CNN kernels in the sparse approach was shown to lead to suboptimal results in case of randomly sampled data [20].

*NW layer* Here the challenge is to adapt the CNN to work around the image sparsity by predicting the missing information. We propose generalisation of NW kernel regression to pCLE image reconstruction from sparse data and their corresponding sparsity mask.

To incorporate NW kernel regression into the CNN framework, we propose a novel trainable CNN layer henceforth referred to as an “NW layer”, which models the relation of the data points by use of custom trainable kernels to perform local interpolation. We define the core NW operation as:

1 $$\begin{aligned} R_{u,v}^{}(S,M)&= \frac{\sum _{i,j=-k}^{k} {S}_{u+i,v+j} w_{i,j} }{\sum _{i,j=-k}^{k} M_{u+i,v+j} | w_{i,j} |}+b \end{aligned}$$

2 $$\begin{aligned} M^{up}&=\sum _{i,j=-k}^{k} M_{u+i,v+j} | w_{i,j} | \end{aligned}$$

The NW layer takes as input *S* and the corresponding known mask *M*. As it propagates through the network, the mask *M* can be seen as a probabilistic sparsity map. Initially $M\in \{0,1\}$, and the next *M* is updated as per Eq. 2 and becomes an approximation of the probability of obtaining reconstructions $R_{u,v}$ given $S_{u,v}$. The $M^{up}$ holds the arbitrary probabilistic sparsity patterns, which are then propagated deeper to the consecutive NW layer. The outputs of the NW layer are reconstructed feature maps *R* estimated using an NW regression and updated probabilistic sparsity masks *M*. Finally, bias *b* is added to *R*. The graphical representation of the NW layer is presented in Fig. 2.

Classical NW kernel regression uses handcrafted, positive kernels. For generalisation of the kernel regression, however, our trainable NW layer allows for negative values. It is necessary for the convolution of the mask *M* to rely on the absolute value of *W*, as this operation is meant to capture the geometric influence of neighbouring pixels on the predicted values of $R_{u,v}$. For numerical stability, we also normalise the kernels such that $\sum _{i,j=-k}^{k} |w_{i,j}|=1$, where *w* is a weight in position *i*, *j* in the *W*.

Thanks to shared kernels between the convolutions used in the NW operation, this layer has the same model complexity as the dense and sparse approaches. Model complexity is indeed measured by the number of parameters or equivalently the number of kernels used. The only addition in terms of complexity is in terms of memory and computation: we keep track of the sparsity pattern and introduce a division of convolutions which essentially is the key to enforcing Nadaraya–Watson regression in CNN framework.

*NWnet framework* Multiple NW layers can be stacked to generalise and benefit NW kernel regression for a irregularly sampled pCLE data. This in turn facilitates end-to-end pipelines that can incorporate sparse inputs by combining NW layers with classical CNNs. As illustrated in Fig. 2, we show how to combine NW layers into deep(er) network. Each NW layer has *t* unique kernels *W*. The first $(n=1)$ layer takes as input *S* and binary *M* and returns *t* feature maps *R* and *t* updated sparsity masks $M_{up}$ which become the input for the next NW layer. The last NW layer of the NWNet framework returns only *t* feature maps *R*, and masks *M* are discarded. NWNet in combination with complex deep learning models, such as EDSR [14], may allow for reconstruction of higher-quality pCLE than the more typically used interpolation.

## Experimental set-up

*Data collection and pre-processing* Synthetic videos were created were created using the methodology from “Data generation methodology for the comparative study” section from high-quality, large histopathological images. We created sets: a training set built with 540 files and a validation set built with 227 files from publicly available histological image data sets [9, 19]. The synthetic test set was created with ten histopathologies from publicly available data called Kather published by [10]. The synthetic test set facilitates making a comparative study between baseline solution, our proposed methodology, and classical CNN solutions.

**Fig. 3:**
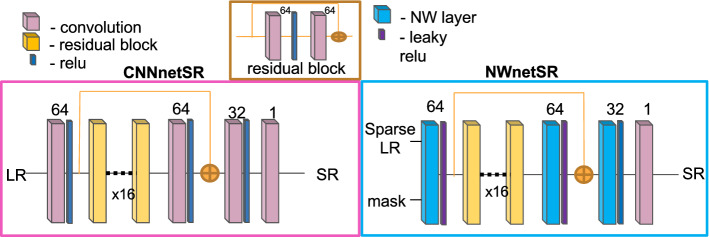
Reconstruction architectures: NWnetSR and CNNnetSR

To facilitate training, video sequences were normalised for each frame individually by subtracting mean and standard deviation of LR frame as follows: $LR = (LR - mean_{LR})/std_{LR}$ and $HR =( HR - mean_{LR})/std_{LR}$, and then scaled to the range [0, 1]. Synthetic LR images were transformed to the synthetic sparse LR images by setting zeros to all pixels which do not correspond to any fibre signal. The masks were generated as binary image, where fibre positions from LR image are set to ones. Lastly to perform batch-based training, we extracted non-overlapping $64 \times 64$ sparse and Cartesian patches for the train and validation sets. The test sets were built with sparse and Cartesian full-size synthetic pCLE images.

*Implementation details* Inspired by EDSR network [14], we design two architectures CNNnetSR and NWnetSR presented in Fig. 3. CNNnetSR was design to perform the SR task from Cartesian or sparse LR pCLE images. The network is very similar to EDSR architecture [14], but with two small improvements. First, we do not use upsampling layer, because synthetic LR and HR have the same size. In place of upsampling layer, we put convolution layer with 32 filters. Second, the last convolutional layer aims at fusing the output feature maps from the penultimate layer into the Cartesian image. We find it more beneficial to use kernel of size one, which is commonly used to reduce number of features maps, than three. All convolutional layers have the kernel size 3. Last layer uses linear activation function.

We want to take advantage from SISR for NW kernel regression, so we design NWnetSR based on CNNnetSR by partly replacing standard convolution with NW layers. For fist NW layer, the kernel size is 9 across each image dimension. The size was chosen based on known distribution of fibres across a Cartesian image to ensure that each convolution would capture more than 10 informative pixels. For deeper NW layers kernel size is 3. The NW weights were initialised with a truncated normal distribution with mean and standard deviation equal to 0.2 and 0.05, respectively.

*Training strategy* To achieve the best training results for each model individually, networks were trained with Population Based Training (PBT) [8]. PBT is it an optimisation technique design to find the best training parameters for the network. During PBT training, a population of the models with different parameters is trained; these models are periodically validated and the weights from best performing model in population are copied to other members of the population. We set the population size to 6 workers, where each member of the population uses a single GPU optimising a network for 1 epochs in one PBT iteration for a total of 100 iterations. The perturbation interval was set for every 20 iterations. The hyperparameter search applied to the 6 learning rates, which were initially set to $10 ^{i}$ where $i\in \{-2,-3,\ldots ,-7\}$. We used the Adam optimiser and set $\beta _{1}$ = 0.9, $\beta _{2}$ = 0.999, $\epsilon =10^{-8}$. Based on results presented in [18], the models were trained with SSIM+L1 loss [24]. Finally, the best performing model from the population is used to generate results on the test sets described in “Data generation methodology for the comparative study” section.

*Experiments* Our baseline methods are: linear interpolation based on Delaunay triangulation referred to LINEAR BASELINE, and NW kernel regression with a crafted single Gaussian kernel referred to GAUSS BASELINE.

**Fig. 4:**
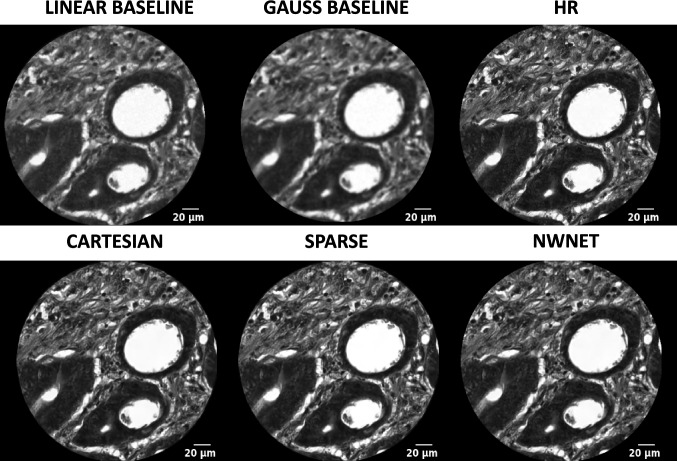
Sample reconstructions from 6 methods handling sparse data in different ways

We compared performance of the DL models: CARTESIAN for CNNnetSR trained with Cartesian input, SPARSE for CNNnetSR trained with sparse input and NWNET for NWnetSR trained with sparse input and corresponding mask. These models were handling irregular signals as input differently.

To quantify how the standard convolution performs on sparse pCLE images in comparison to using Cartesian reconstructions as the input, we trained two unique models based on CNNnetSR network: CARTESIAN trained using reconstructed Cartesian images and SPARSE one trained with sparse images.

To test NW kernel regression benefits from generalisation via learning multiple kernels, we trained the NWnetSR as EBSR network for the task of pCLE SR reconstruction with sparse input images and masks.

## Results

The final performance of the models is evaluated by comparing quality of the reconstructed SR pCLE with the HR synthetic images from the test set. To measure the image quality of the SR pCLE reconstructions, we design an image quality assessment (IQA) procedure which consists of two complementary metrics typically used for this task: peak signal-to-noise ratio (PSNR) and the structural similarity index SSIM [23].

The results computed for the images from the test set, described in “Data generation methodology for the comparative study” section, are shown in Table 1. The main observation is that each DL model outperforms the baseline interpolation technique. Training of the NWnetSR as SISR network generalises NW kernel regression and outperforms traditional NW regression with handcrafted Gaussian kernel, proving that use of many kernels which are estimated on the data set is more beneficial then custom single Gaussian kernel. There is no significant improvement for PSNR and SSIM scores when comparing DL models between each other. They perform almost indistinguishably. We also provide example reconstructions in Fig. 4, and it can be noticed easily that all DL images are almost identical.

**Table 1:** IQA: comparison on average performance of 5 methods aimed at pCLE image reconstructions. Table shows average PSNR and SSIM score with standard deviation for all frames in 10 pseudo pCLE videos

Model name	SSIM	PSNR
GAUSS BASELINE	0.758 ± 0.025	25.88 ± 1.0
LINEAR BASELINE	0.800 ± 0.018	24.65 ± 1.2
CARTESIAN	0.902 ± 0.015	30.98 ± 1.1
SPARSE	0.907 ± 0.016	30.99 ± 1.0
NWNET	0.900 ± 0.015	30.96 ± 1.1

We analysed qualitatively SR reconstructions, and we can observe two tendencies for all DL models. First, SR reconstructions differ slightly on a pixel level, but that differences do not affect how image is perceived as whole, and may be a reason for slightly different metrics score during IQA. Second, in the opinion of our clinical collaborators, SR reconstructions are clearly more aesthetically pleasing than baseline reconstructions LINEAR BASELINE and GAUSS BASELINE. Neither of SR has triangulation artefacts, and every SR reconstruction has significantly reduced noise, additionally benefiting from improved contrast and visibility of details.

The results confirm that the NW layer among other layers handling sparse data is an choice for image reconstruction and yields increasingly good image-quality results from sparse pCLE data to Cartesian SR image.

## Discussion and conclusions

In this research work, we focused on providing a comparative study of learning-based approaches for pCLE super-resolution. In the context of pCLE, this is the first work which proposes end-to-end deep learning image reconstructions from irregularly sampled fibre data and provides a head-to-head comparison of sparse and Cartesian approaches for this task.

We proposed NW layer which enables the use of sparse images as input and performs deep generalised Nadaraya–Watson kernel regression. We have shown the successful implementation of the reconstruction pipeline, which combines NW and CNN layers and is trained in a supervised manner as SISR, reconstructing super-resolved images from sparse input images.

We demonstrated that learning-based methods reconstruct pCLE images with improved image quality. Our results show that these methods outperform baseline solutions. Yet, no significant differences between deep methods were found. Although somewhat negative, we believe this result is important because of its counter-intuitive nature.

The observation that all DL approaches perform equally well with no statistically significant difference may mean that standard deep CNNs are powerful enough to solve the reconstruction of sparse pCLE images regardless of their sparsity. A potential reason for it may lie in the fact that fibres in the pCLE bundle are distributed in a pseudo-regular pattern (quasi-hexagonal). This pseudo-regularity is probably key to steer the Cartesian and sparse model towards a suitable solution. Previous related studies on randomly distributed LIDAR data, which do not display the same pseudo-regularity pattern, showed that a sparse approach that explicitly accounts for data sparsity is helpful. The sparse learning problem may indeed be more complex in such cases than in the case of pCLE data.

## Electronic supplementary material

Below is the link to the electronic supplementary material.

Supplementary material 1 (pdf 4052 KB)

Supplementary material 2 (avi 5257 KB)

Supplementary material 3 (avi 3577 KB)

Supplementary material 4 (avi 3192 KB)

Supplementary material 5 (avi 7075 KB)

Supplementary material 6 (avi 6542 KB)
