# Supplementary material for: Learning from irregularly sampled data for endomicroscopy super-resolution: a comparative study of sparse and dense approaches
Source: Int J Comput Assist Radiol Surg. 2020 May 15;15(7):1167–75. doi: 10.1007/s11548-020-02170-7 (PMC7316691; doi:10.1007/s11548-020-02170-7)
Supplement: Supplementary file 1 — Supplementary material 1 (pdf 4052 KB) [file 11548_2020_2170_MOESM1_ESM.pdf]

Supplementary materials: Learning from Irregularly Sampled Data for Endomicroscopy Super-resolution: A Comparative Study of Sparse and Dense Approaches

Agnieszka Barbara Szczotka Dzhoshkun Ismail Shakir Daniele Ravì Matthew J. Clarkson
Stephen P. Pereira Tom Vercauteren

March 2020

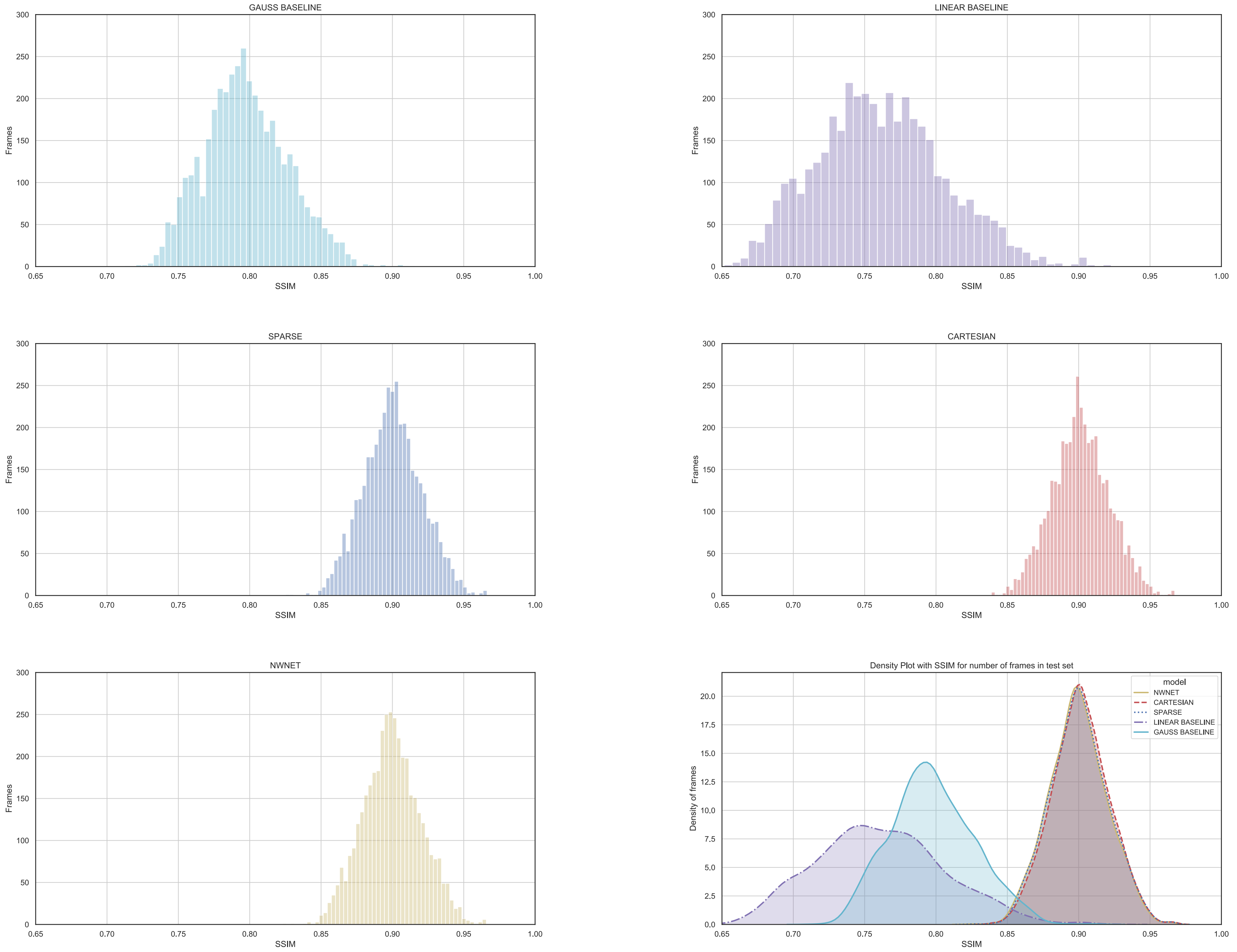

Figure 1: SSIM error analysis for test set. Five histograms of SSIM score, one for each model; and the normalised density plot comparing all models together.

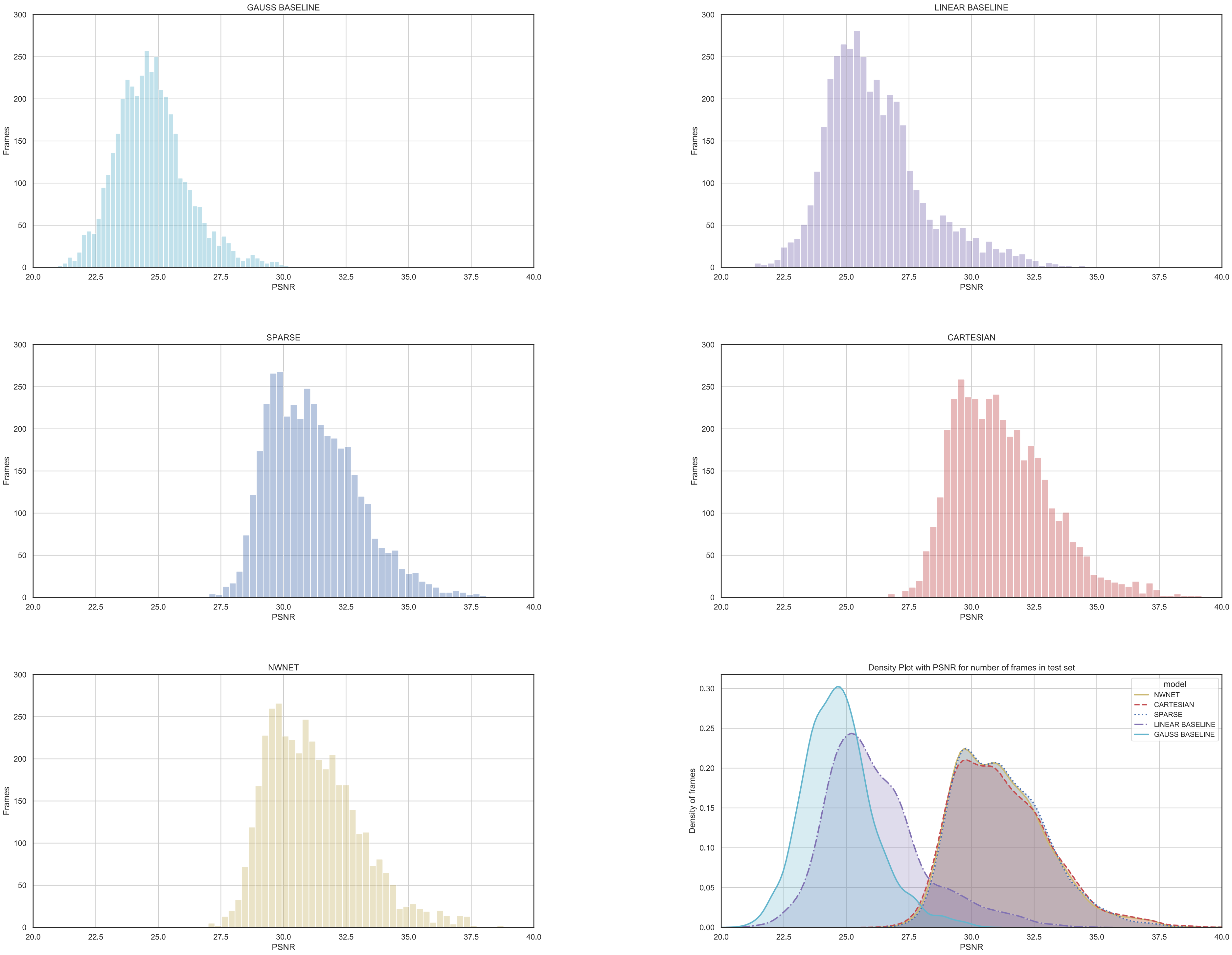

Figure 1: PSNR error analysis for test set. Five histograms of PSNR score, one for each model; and the normalised density plot comparing all models together.

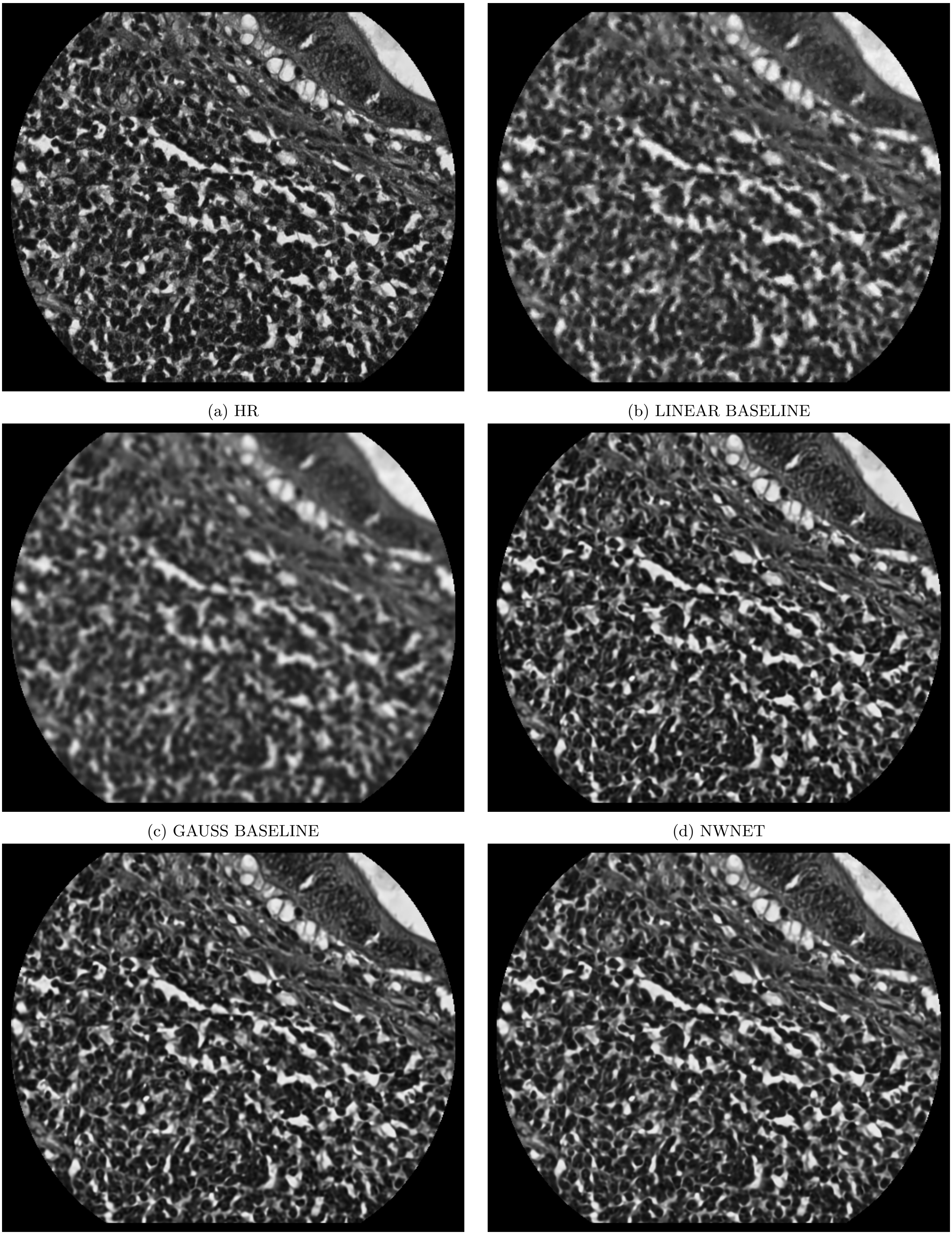

(a) HR

(b) LINEAR BASELINE

(c) GAUSS BASELINE

(d) NWNET

(e) CARTESIAN

(f) SPARSE

Figure 1: Frames with the lowest PSNR score in the test set for each model.

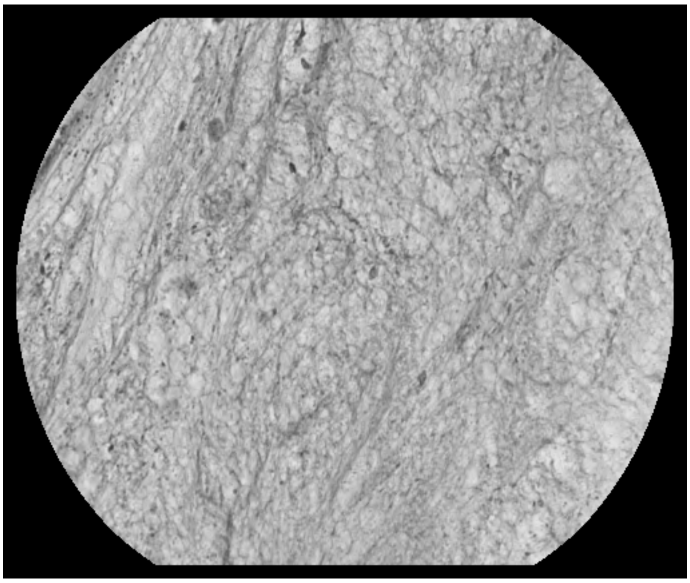

(a) HR

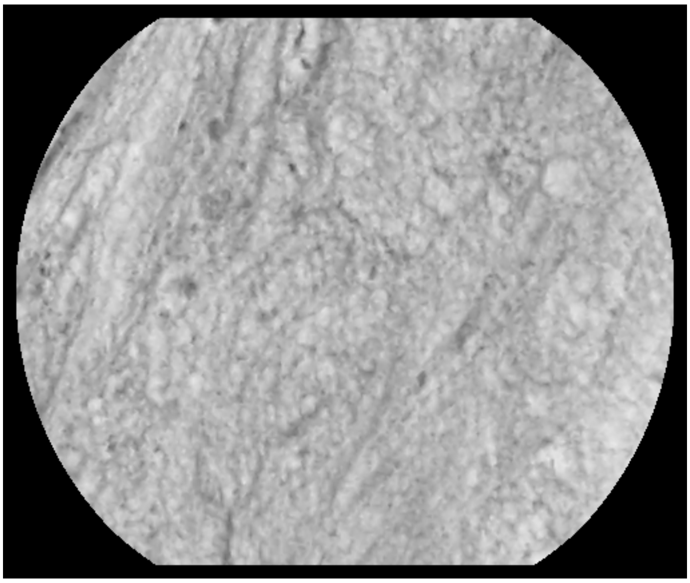

(b) LINEAR BASELINE

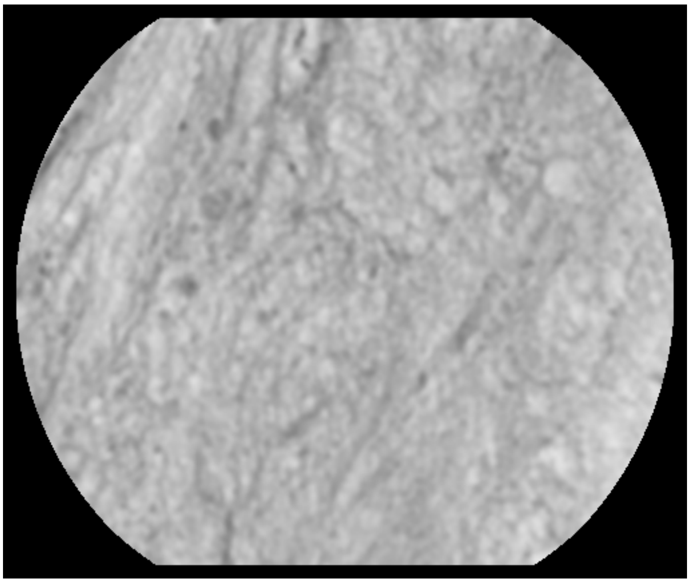

(c) GAUSS BASELINE

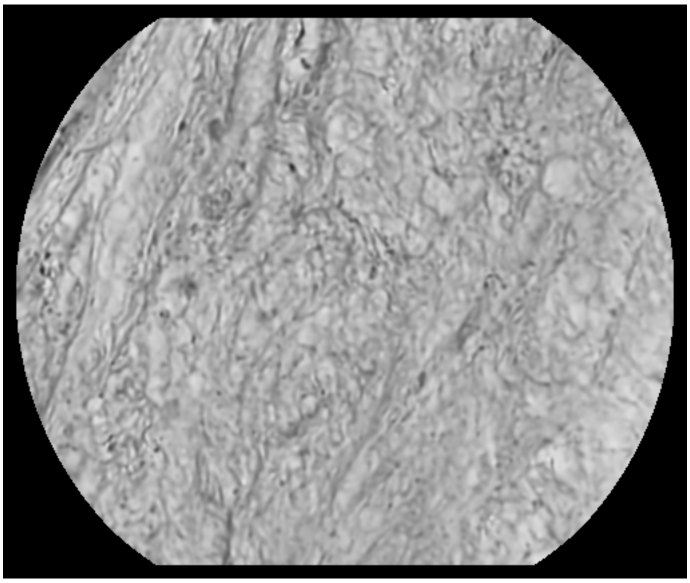

(d) NWNET

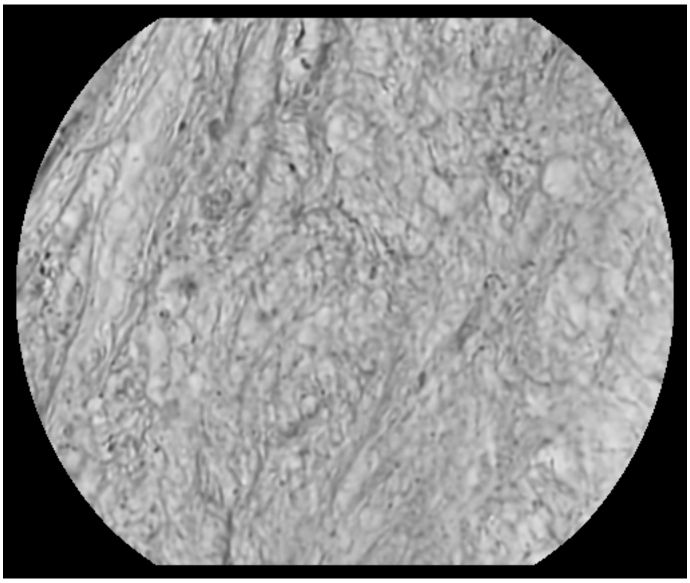

(e) CARTESIAN

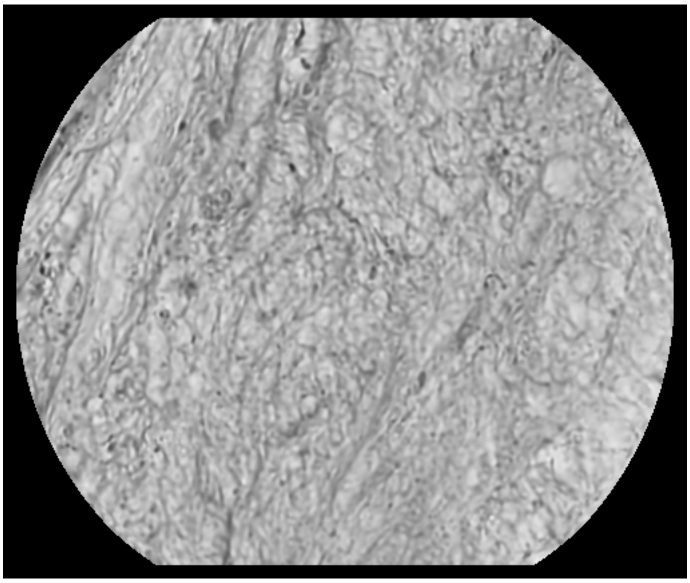

(f) SPARSE

Figure 2: Frames with the lowest SSIM score in the test set for each model.

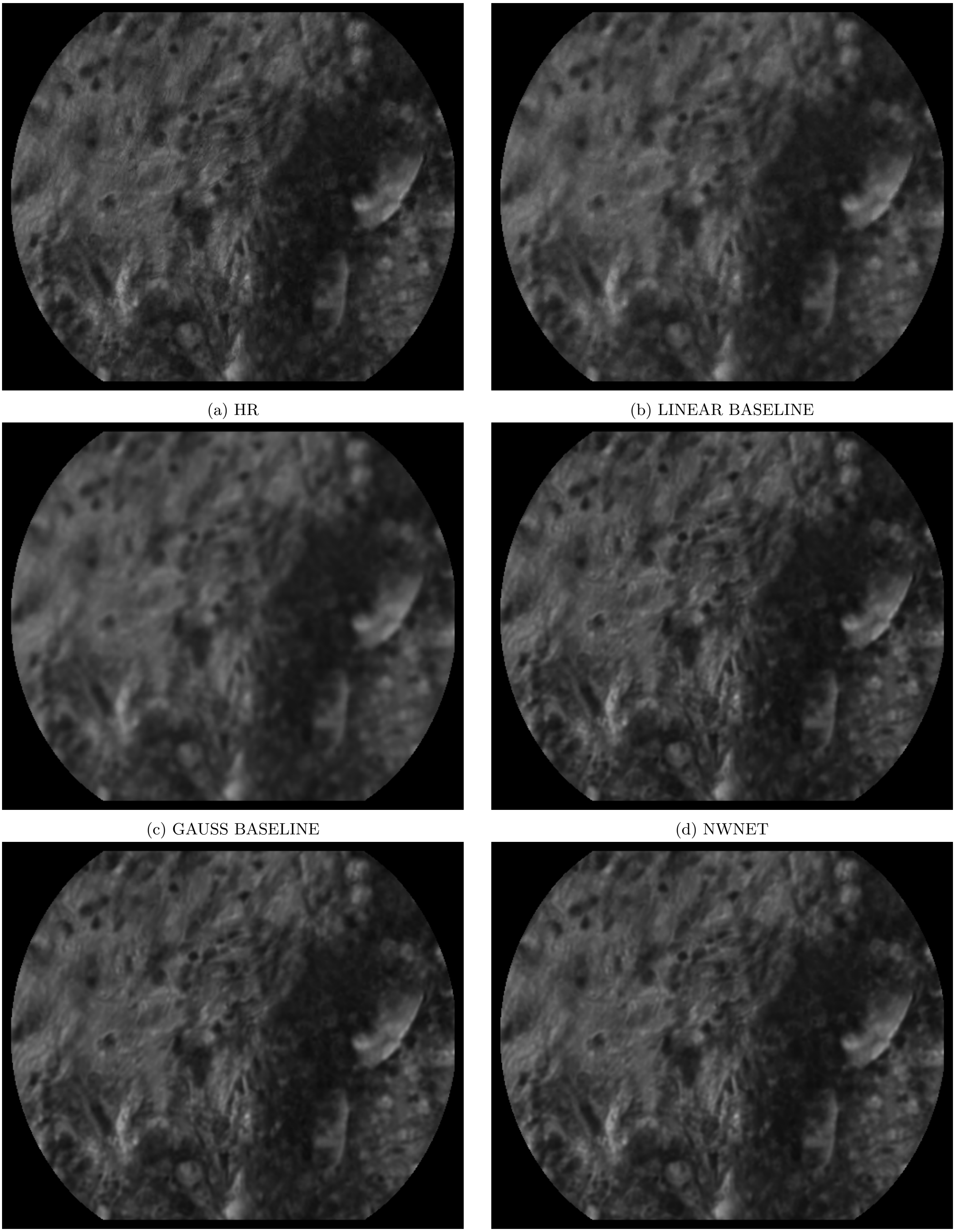

(a) HR

(b) LINEAR BASELINE

(c) GAUSS BASELINE

(d) NWNET

(e) CARTESIAN

(f) SPARSE

Figure 3: Frames with the highest PSNR and SSIM score in the test set for each model. These frames also represent the biggest improvement in PSNR score in reference to the LINEAR BASELINE.

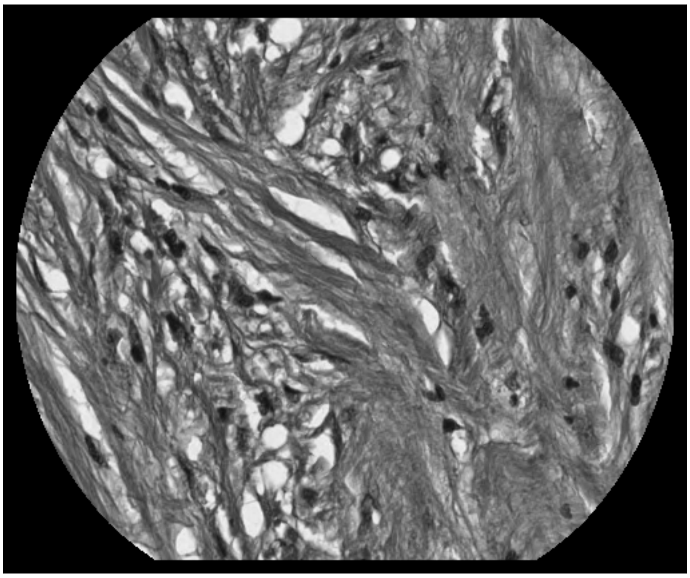

(a) HR

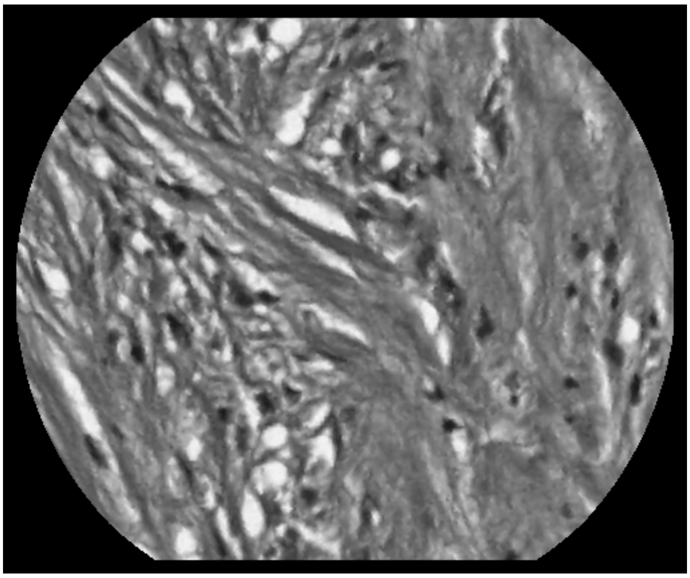

(b) LINEAR BASELINE

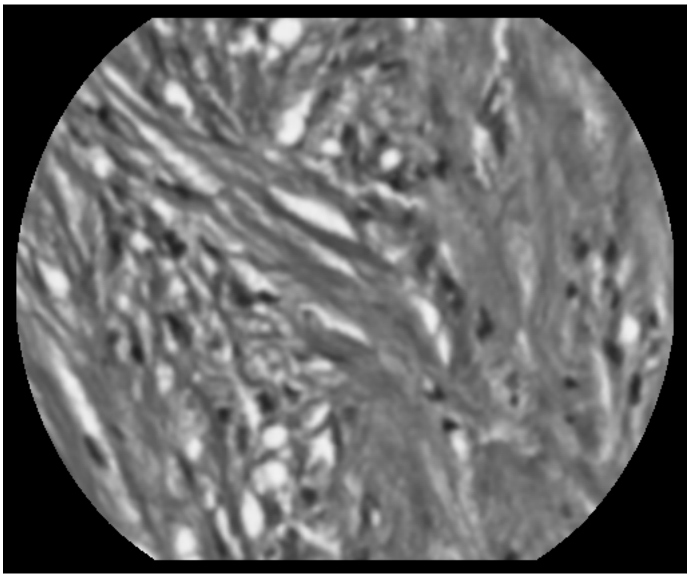

(c) GAUSS BASELINE

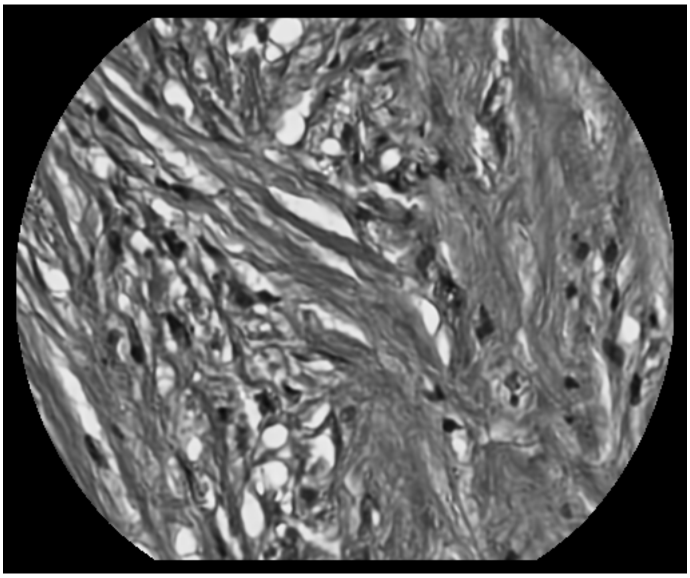

(d) NWNET

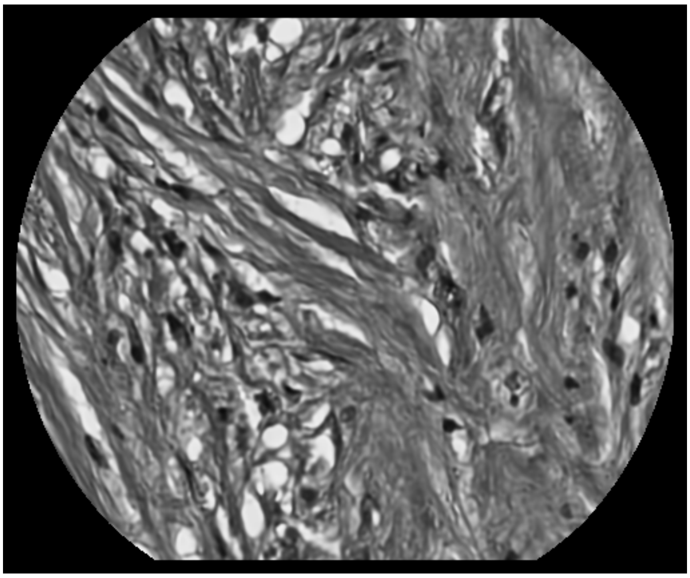

(e) CARTESIAN

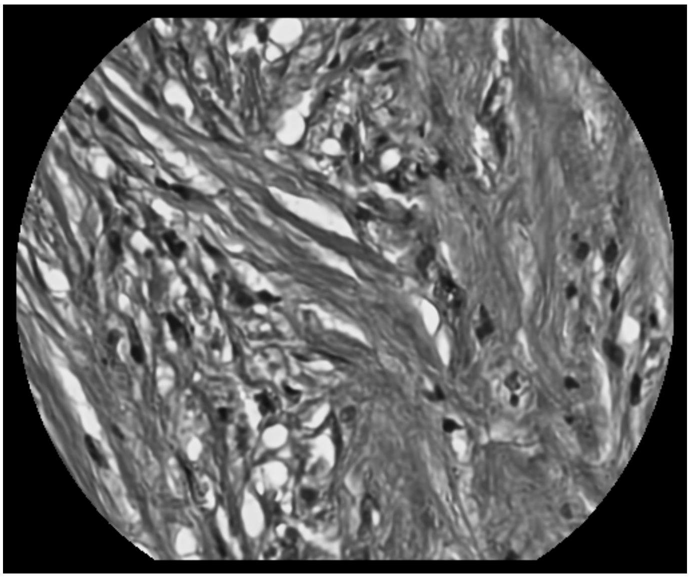

(f) SPARSE

Figure 4: Frames with the smallest improvement in PSNR score in reference to the LINEAR BASELINE in the test set for each model.

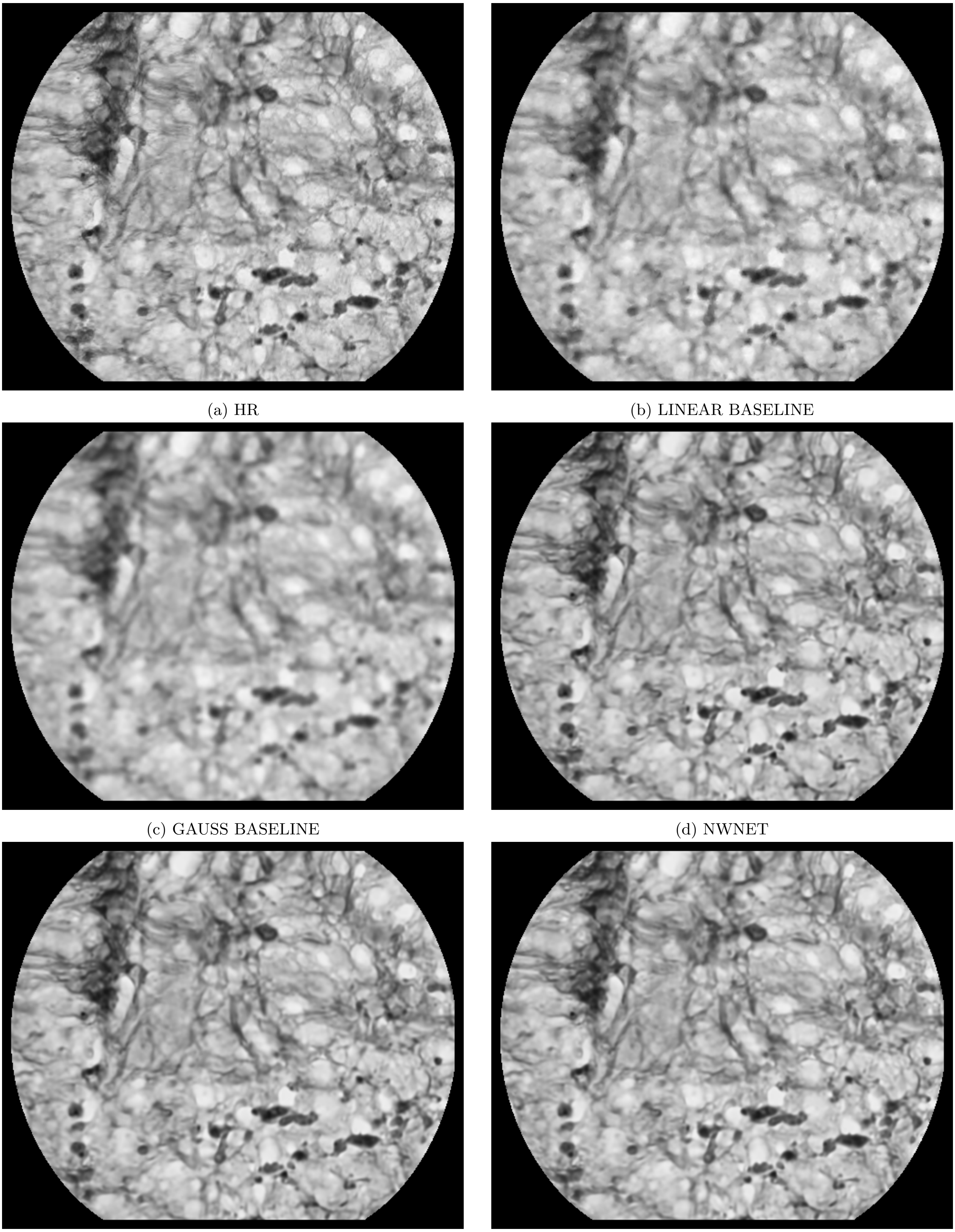

(a) HR

(b) LINEAR BASELINE

(c) GAUSS BASELINE

(d) NWNET

(e) CARTESIAN

(f) SPARSE

Figure 5: Frames with the biggest improvement in SSIM score in reference to the LINEAR BASELINE in the test set for each model.

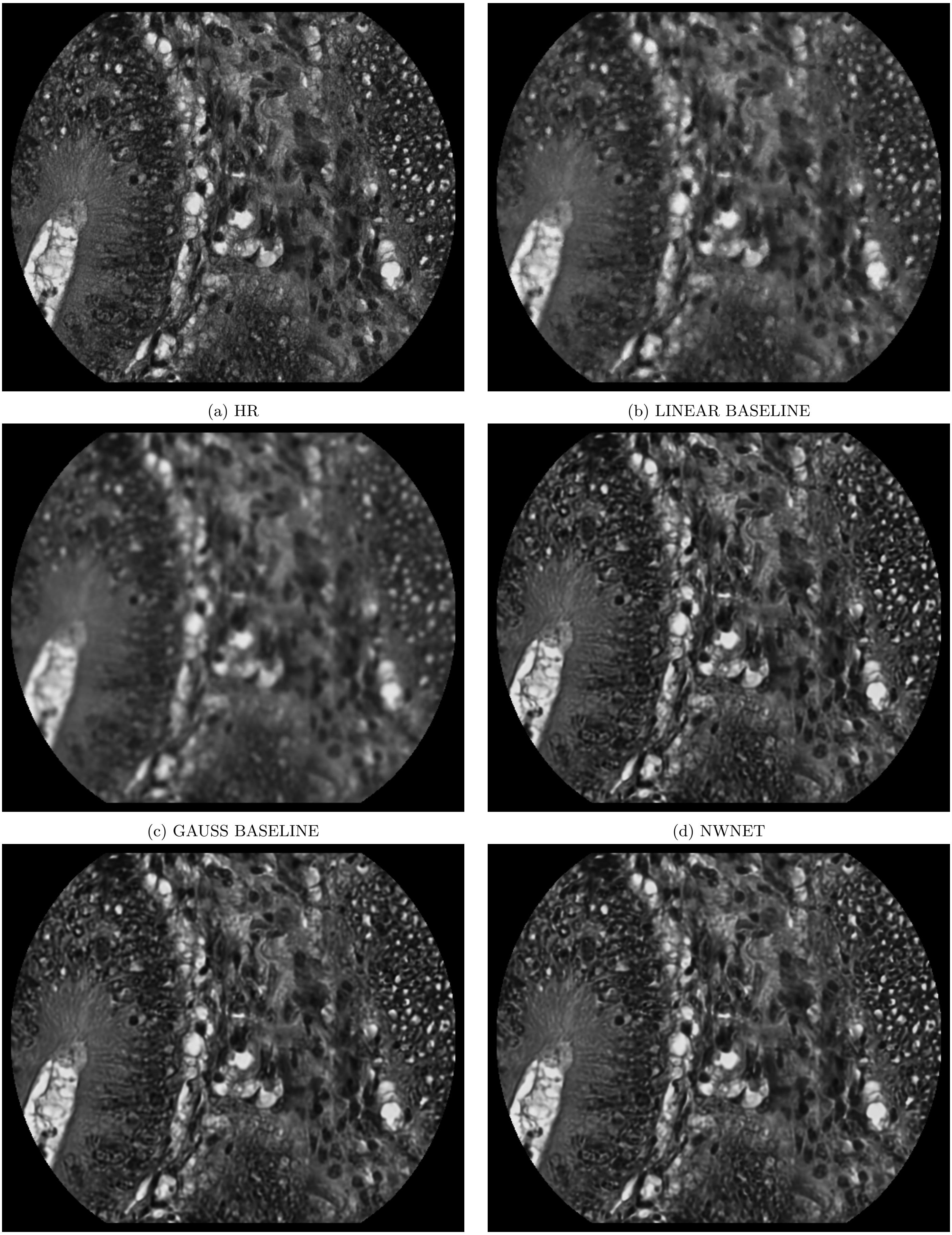

(a) HR
(b) LINEAR BASELINE
(c) GAUSS BASELINE
(d) NWNET
(e) CARTESIAN
(f) SPARSE

Figure 6: Frames with the smallest improvement in SSIM score in reference to LINEAR BASELINE in test set for each model.
